# Unexpected Binding Mode of a Potent Indeno[1,2-*b*]indole-Type Inhibitor of Protein Kinase CK2 Revealed by Complex Structures with the Catalytic Subunit CK2α and Its Paralog CK2α′

**DOI:** 10.3390/ph10040098

**Published:** 2017-12-13

**Authors:** Jennifer Hochscherf, Dirk Lindenblatt, Benedict Witulski, Robin Birus, Dagmar Aichele, Christelle Marminon, Zouhair Bouaziz, Marc Le Borgne, Joachim Jose, Karsten Niefind

**Affiliations:** 1 Department für Chemie, Institut für Biochemie, Universität zu Köln, Zülpicher Straße 47, D-50674 Köln, Germany; j.hochscherf@uni-koeln.de (J.H.); dlinden0@smail.uni-koeln.de (D.L.); benedict.witulski@gmx.de (B.W.); 2 Institut für Pharmazeutische und Medizinische Chemie, PharmaCampus, Westfälische Wilhelms-Universität Münster, Corrensstraße 48, D-48149 Münster, Germany; robin.birus@uni-muenster.de (R.B.); dagmar.aichele@uni-muenster.de (D.A.); joachim.jose@uni-muenster.de (J.J.); 3 EA4446 Bioactive Molecules and Medicinal Chemistry, SFR Santé Lyon-Est CNRS UMS3453-INSERM US7, Faculté de Pharmacie—ISPB, Université Claude Bernard Lyon 1, 8 avenue Rockefeller, F-69373 Lyon CEDEX 8, France; christelle.marminon-davoust@univ-lyon1.fr (C.M.); zouhair.bouaziz@univ-lyon1.fr (Z.B.); marc.le-borgne@univ-lyon1.fr (M.L.B.)

**Keywords:** protein kinase CK2, casein kinase 2, paralogous isoforms CK2α and CK2α′, indeno[1,2-*b*]indole scaffold, membrane permeability, ATP-competitive inhibitors, dual inhibitors

## Abstract

Protein kinase CK2, a member of the eukaryotic protein kinase superfamily, is associated with cancer and other human pathologies and thus an attractive drug target. The indeno[1,2-*b*]indole scaffold is a novel lead structure to develop ATP-competitive CK2 inhibitors. Some indeno[1,2-*b*]indole-based CK2 inhibitors additionally obstruct ABCG2, an ABC half transporter overexpressed in breast cancer and co-responsible for drug efflux and resistance. Comprehensive derivatization studies revealed substitutions of the indeno[1,2-*b*]indole framework that boost either the CK2 or the ABCG2 selectivity or even support the dual inhibition potential. The best indeno[1,2-*b*]indole-based CK2 inhibitor described yet (IC_50_ = 25 nM) is 5-isopropyl-4-(3-methylbut-2-enyl-oxy)-5,6,7,8-tetrahydroindeno[1,2-*b*]indole-9,10-dione (**4p**). Herein, we demonstrate the membrane permeability of **4p** and describe co-crystal structures of **4p** with CK2α and CK2α′, the paralogs of human CK2 catalytic subunit. As expected, **4p** occupies the narrow, hydrophobic ATP site of CK2α/CK2α′, but surprisingly with a unique orientation: its hydrophobic substituents point towards the solvent while its two oxo groups are hydrogen-bonded to a hidden water molecule. An equivalent water molecule was found in many CK2α structures, but never as a critical mediator of ligand binding. This unexpected binding mode is independent of the interdomain hinge/helix αD region conformation and of the salt content in the crystallization medium.

## 1. Introduction

Protein kinase CK2 (former name: casein kinase 2), a serine/threonine kinase of the CMGC subgroup of eukaryotic protein kinases (EPKs) [1], is involved in cellular key processes like cell cycle progression [2], evasion of apoptosis [3] and DNA damage repair [4]. CK2 counteracts pro-apoptotic stimuli and promotes cell proliferation. Abnormally high levels of CK2 catalytic activity or mRNA have been reported for tumour cells and tissues [5,6,7]. Accordingly, CK2 is associated to several types of cancer—among them glioblastoma [8], pancreatic cancer [9] and leukaemia [10]—and to a variety of other human pathologies [11] such as neurodevelopmental [12] and neurodegenerative [13] diseases and diabetes [14]. Therefore, the enzyme is subject of intensive efforts of inhibitor design in order to target either the ATP-binding site [15,16] or exosites [17] or two binding sites simultaneously [18,19,20,21].

Structurally CK2 predominantly occurs as a heterotetrameric complex [22] with a central dimer of non-catalytic subunits (CK2β) to which two catalytic subunits (CK2α) are attached [23]. This so-called “CK2 holoenzyme” is prone to reversibly form linear or circular aggregates and in a salt-dependent manner [24,25], but the physiological and regulatory significance of this feature is unclear [26,27].

For the catalytic subunit in humans and other vertebrates, two paralogous isoforms exist. They are typically denoted as CK2α and CK2α′ and have highly similar sequences with exception of their completely unrelated C-terminal segments [28]. *CSNK2A1* and *CSNK2A2*, the genes coding for the two isozymes, are located on different chromosomes [28]. The paralogs have similar enzymatic characteristics, and are expressed in all tissues according to the Human Protein Atlas [29]; neverthe-less, the mere existence of paralogs suggests a functional differentiation between CK2α and CK2α′ [30]. In fact, a number of features are consistent with this assumption: (i) the C-terminus of CK2α′ lacks phosphorylation sites, but they are present and phosphorylated in a cell-cycle-dependent manner in CK2α [2]; (ii) the affinity of CK2α′ and CK2β is lower than the affinity between CK2α and CK2β [31] and the aggregation propensities of the resulting holoenzymes differ as well [32,33]; (iii) the proteins CKIP-1 [30,34] and PP2A [35] are isoform-specific binding partners of CK2α; (iv) while a knockout of CK2α in mice is embryonically lethal [36], a knockout of CK2α′ just leads to an impaired spermatogenesis [37]. The latter observation indicates that CK2α can compensate the loss of CK2α′ to a large extend [30]. This reflects a kind of priority for this isoform that may be one reason why the majority of studies focusses on CK2α rather than CK2α′. This tendency is also obvious in structural analyses: while more than 90 structures of human CK2α (including CK2 holoenzyme structures) have been deposited in the Protein Data Bank (PDB), only 4 structures of human CK2α′ are available (PDB codes 5M4U [38], 5M56 [38], 3OFM [31], 3E3B [39]). The large majority of CK2α structures and all CK2α′ structures are complex structures with various ATP-competitive inhibitors.

The most important common feature of ATP-competitive CK2-inhibitors is a flat and hydrophobic, predominantly aromatic framework. It is complementary to an ATP-site which is—compared to other EPKs—rather narrow and hydrophobic due to some bulky and non-polar side chains located at both flanking domains [40]. However, with respect to the peripheral derivatization with halogens, charged and aromatic groups and hydrogen bond donors/acceptors a fairly broad variety of substituents was found in the past [41]. Many CK2 inhibitors—among them CX-4945 (silmitasertib) [42] which acquired the state of a “benchmark” inhibitor in recent years—form electrostatic interactions with a positively charged area near Lys68 [43] and π-π-interactions with Phe113 [44], CK2α′s equivalent of the critical “gatekeeper” residue of EPKs [45]. Furthermore, an important anchor region for hydrogen/halogen bonds is the peptide backbone of the interdomain hinge. Emodin and other examples have shown that CK2 inhibitors do not necessarily require these hinge interactions for binding [46], but to achieve affinities in the low nanomolar range they are regarded as imperative [41].

**Figure 1:**
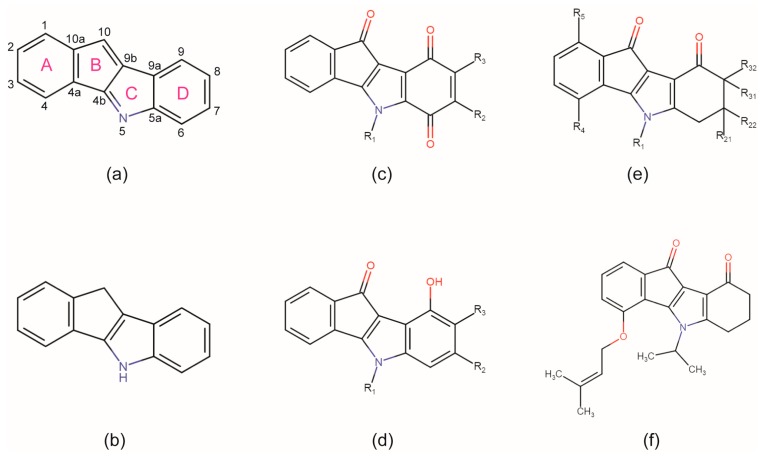
The indeno[1,2-*b*]indole framework with atom and ring labels (**a**), its reduced form (**b**) and some of its substitution possibilities to create effective CK2 inhibitors. (**c**–**e**) The quinonic (**c**), phenolic (**d**) and ketonic (**e**) cluster of indeno[1,2-*b*]indole-type CK2 inhibitor candidates as defined by Haidar et al. [56]. (**f**) The CK2 inhibitor 5-isopropyl-4-(3-methylbut-2-enyloxy)-5,6,7,8-tetrahydro-indeno[1,2-*b*]indole-9,10-dione (**4p**), described by Gozzi et al. [52] and used for the experiments of this study.

The indeno[1,2-*b*]indole scaffold (Figure 1a)—in particular the reduced form 5,10-dihydro indeno[1,2-*b*]indole (Figure 1b)—is a relatively new member among CK2 targeting lead structures. Molecules derived from this framework have been known as pharmacologically relevant in the context of tumour therapy for a long time [47,48]. Bal et al. [49] described five indeno[1,2-*b*]indole derivatives that intercalate DNA, stimulate the DNA-breaking activity of DNA-topoisomerase II and are thus strongly cytotoxic for human leukemia cells.

A particularly attractive observation of Bal et al. [49] was the fact that their indeno[1,2-*b*]indole compounds are only weakly affected by drug efflux. This suggested interesting off-target effects with multi-drug resistance transport systems typically induced under cytotoxic conditions. This feature, further the flat and extended hydrophobic scaffold that resembled ATP-competitive CK2 inhibitors [41], and finally the plethora of functionalization opportunities provided by a tetracyclic ring system inspired ideas to use the indeno[1,2-*b*]indole framework as the basis for polypharma-cology approaches: first Hundsdörfer et al. [50,51] described a collection of indeno[1,2-*b*]indole-type CK2 inhibitors all of them equipped with an oxo group at position 10 (Figure 1c–e) and the best of them with an attractive selectivity profile against a panel of 22 EPKs; later Gozzi et al. [52,53] demonstrated how this indeno[1,2-*b*]indole-10-one scaffold can be further derivatized at the rings A, C and D in different ways in order to create inhibitors targeting selectively either CK2 or the breast cancer resistance protein ABCG2—an ABC half transporter overexpressed in breast cancer cells [54]; and finally Alchab et al. [55] extended this differentiation to a third cancer-relevant enzyme target, namely the cell cycle key phosphatase CDC25. In summary, with respect to the target CK2 about 50 indeno[1,2-*b*]indole-based inhibitor candidates were described which have been recently clustered according to their D-ring substitution into a quinonic (Figure 1c), a phenolic (Figure 1d) and a ketonic (Figure 1e) subgroup [56]. 

In spite of the wealth of knowledge about effects of indeno[1,2-*b*]indole-type molecules on various enzymes the structural bases of these functionalities are largely unknown so far. A survey in the Protein Data Bank (PDB) with either the indeno[1,2-*b*]indole framework (Figure 1a) or with its 5,10-dihydro derivative (Figure 1b) using the small molecule search routines of www.rcsb.org [57] did not provide a single hit—neither in the “substructure” mode nor in the “similarity” mode down to a similarity grade of 50%. This means that no indeno[1,2-*b*]indole-type ligand has been deposited in the PDB to date.

Due to this lack of structural knowledge Alchab et al. [58] created in-silico 3D-models of four substituted indeno[1,2-*b*]indoles in complex with human CK2α in order to rationalize experimentally determined SAR results. We will compare a representative of these models here with the first X-ray structures of an indeno[1,2-*b*]indole derivative in complex with human CK2α or CK2α′ which we determined with 5-isopropyl-4-(3-methylbut-2-enyloxy)-5,6,7,8-tetrahydro-indeno[1,2-*b*]indole-9,10-dione (Figure 1f), the best indeno[1,2-*b*]indole-type CK2 inhibitor described so far and a result of the aforementioned CK2-ABCG2 cross-linked work of Gozzi et al. [52] who referred to this compound as **4p**. **4p** does not possess the typical anchor groups of high-affinity CK2 inhibitors, like a carboxy group, halogen atoms for halogen bonding or hydrogen bond donors (Figure 1f), making its low IC_50_-value of 25 nM [52] insofar particularly remarkable and pointing towards an unusual binding mode that awaits clarification by X-ray crystallography.

## 2. Results and Discussion

### 2.1. Membrane Permeability of the Indeno[1,2-*b*] Indole Compound ***4p***

**Figure 2:**
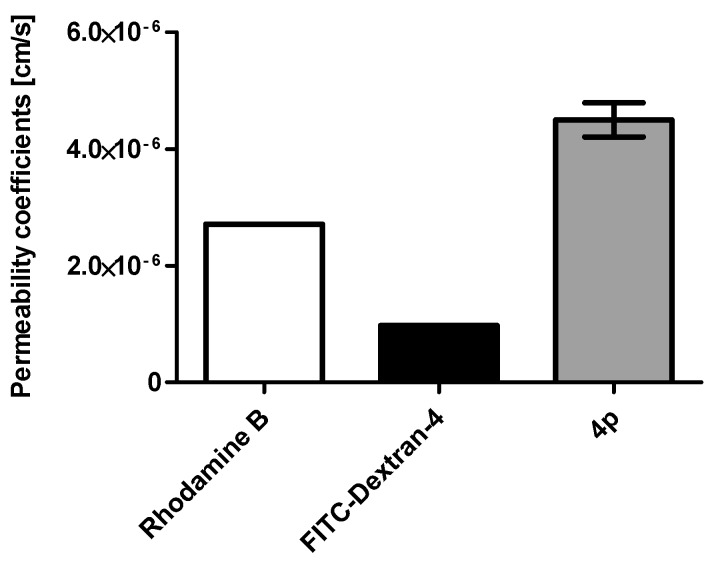
Determination of the cell permeability of **4p** using a Caco-2 assay based on human epithelial colecteral adenocarcinoma cells. Rhodamine B served as a positive control and FITC-dextran 4 as a negative control.

The ability of **4p** to enter cells was already indirectly demonstrated by Gozzi et al. [52] who determined significant cytotoxic and anti-proliferative effects of the compound. To confirm and supplement these results we quantified the membrane permeability of **4p** by measuring its apparent permeability coefficient P_app_ using human epithelial colorectal adenocarcinoma cells (Caco-2) in a Caco-2 assay [59,60]. We determined a P_app_-value of 4.46 × 10^−6^ cm/s which is almost five times higher than that of the negative control FITC-labeled dextran-4 (9.71 × 10^−7^ cm/s) and even higher than the value of the positive control rhodamine B (2.71 × 10^–6^ cm/s) (Figure 2). This result is fully consistent to the cellular data reported previously [52]. It should be mentioned that indeno[1,2-*b*]indoles as exemplified here by compound **4p** can be absorbed without problems by human cells, which is the most important prerequisite for the development of an orally applicable drug. 

### 2.2. Overview of CK2α/CK2α′ Co-Crystal Structures with the Inhibitor ***4p***

To clarify the structural bases of **4p**’s inhibitory effect on CK2 we determined complex structures of the compound with functional variants of the two isoforms of the human catalytic CK2 subunit, namely with CK2α^1−335^ [61] and with CK2α′^Cys336Ser^ [31]. In both cases efforts were undertaken to crystallize the enzyme/inhibitor complex both under high- and under low-salt conditions in order to investigate any bias of the ionic strength in the crystallization medium on either the local protein conformation or the inhibitor binding mode as observed with other CK2 inhibitors [38,62]. In fact, the CK2α^1−335^/**4p** complex could be crystallized under conditions with strongly differing salt concentrations while the CK2α′^Cys336Ser^/**4p** complex crystallized only under low-salt conditions (Table 1). 

**Figure 3:**
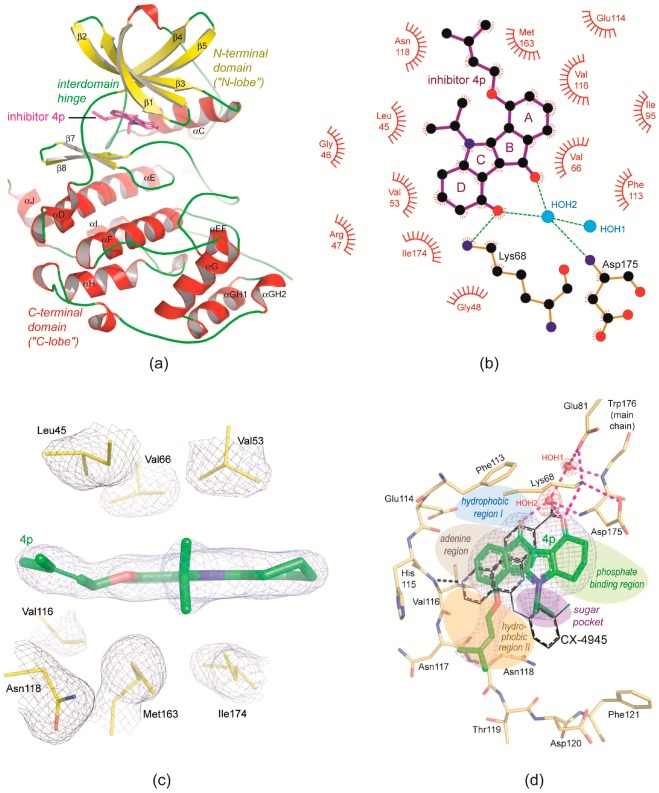
The principle binding mode of **4p** to protein kinase CK2. (**a**) Overview of the CK2α/**4p** complex structure. (**b**) 2D-projection of the non-covalent interactions between **4p** and CK2α. (**c**) Hydrophobic packaging of **4p** by non-polar side chains from the N-lobe (Leu45, Val53 and Val66), from the C-lobe (Met163 and Ile174) and from the interdomain hinge (Val116); the pieces of electron density were drawn with a cutoff level of 1 σ. (**d**) The ATP site of CK2α with bound **4p** embedded in electron density (cutoff level 1 σ); for comparison the CK2 inhibitor CX-4945 was drawn with black C-atoms after superimposition of the protein matrices; the five regions of the ATP-site according to the protein kinase pharmacophore model of Traxler and Furet [63] are indicated by coloured patches. All parts of the figure were prepared with chain A of the low-salt CK2α^1−335^/**4p** structure (no. 2 of Table 1).

Each of the three complex crystals diffracted X-rays to about 2 Å resolution so that complex structures of good quality could be determined (Table 1). In the two low-salt structures (no. 2 of Table 1 with CK2α^1−335^ and no. 3 of Table 1 with CK2α′^Cys336Ser^) two protomers per asymmetric unit are present, respectively, so that the three structures altogether contain five crystallographically independent copies of CK2α^1−335^ or CK2α′^Cys336Ser^. Each of them harbours a **4p** molecule well defined by electron density at the expected location, namely occupying the ATP site at the interface between the two main domains of the kinase core (Figure 3a).

### 2.3. Principle Binding Mode of ***4p*** to the ATP-Site of CK2α/CK2α′

The orientation of **4p** relative to the enzyme and the most important protein/inhibitor inter-actions are identical in all five crystallographically independent enzyme/**4p** complexes. Therefore, for the following description of the binding mode the chain A of the low-salt CK2α^1−335^/**4p** complex (no. 2 of Table 1) was chosen as a representative. The binding of **4p** mainly depends on hydrophobic interactions between the aromatic four-ring system and suitable side chains of the enzyme (Figure 3b,c). The N-lobal β-sheet contributes four side chains from different β-strands (Leu45 from β1, Val53 from β2, Val66 from β3 and Ile95—not drawn in Figure 3c—from β4) to this hydrophobic packaging, while two side chains (Met163 and Ile 174) are from the C-lobe and two (Val116 and the methylene group of Asn118) from the interconnecting hinge (Figure 3c). Noteworthy, the “gatekeeper”-equivalent residue [45] Phe113 is only peripherally involved in this hydrophobic cluster; in particular, in contrast to CK2 inhibitors like CX-4945 [44] (Figure 3d), emodin [46], E9 [64], FLC21 [38] or FLC26 [62] **4p** does not supply an aromatic ring as a partner of a π-π-interaction with Phe113.

**Figure 4:**
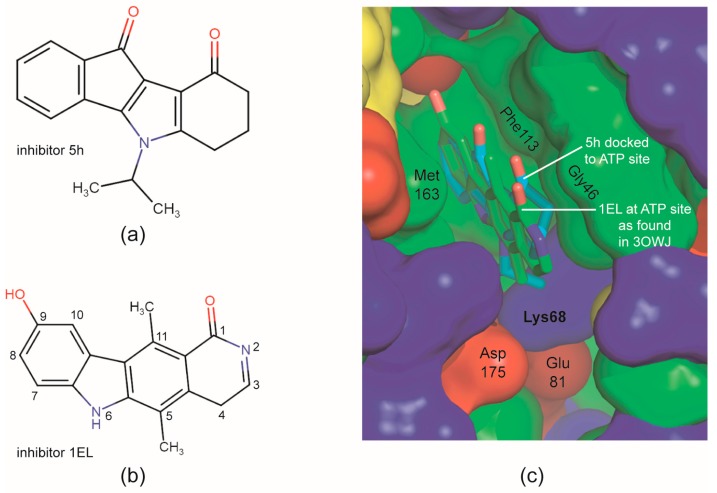
*In silico* structure modelling of CK2α complexes with indeno[1,2-*b*]indole-type CK2 inhibitors according to Alchab et al. [58]. (**a**) 5-Isopropyl-5,6,7,8-tetrahydro-indeno[1,2-*b*]indole-9,10-dione (**5h**), one of four indeno[1,2-*b*]indole-based inhibitors modelled into the ATP-site of CK2α by Alchab et al. [58]. (**b**) The pyridocarbazolone-type CK2 inhibitor 1EL that was co-crystallized with CK2α to give the complex structure 3OWJ [66] which served as a template for modelling of the CK2α/**5h** complex by Alchab et al. [58]. (**c**) Overlay of the X-ray structure of the CK2α complex with 1EL (green C-atoms; PDB 3OWJ) and the *in-silico* model of the CK2α complex of **5h** (light blue C-atoms). Modified version of a picture originally published by Alchab et al. [58] with kind permission of MDPI AG, Basel, Switzerland.

Both the prenyl alcohol group attached to ring A and the isopropyl substituent of ring C participate significantly in these hydrophobic interactions: the former is located in the “hydrophobic region II”, while the latter—which in all five **4p** copies of this study displays the conformation visible in Figure 3c, namely with the two methyl groups being in maximal distance to the indeno[1,2-*b*]indole plane and to the prenyl alcohol substituent—occupies the “sugar pocket” according to a well-established pharmacophore model for EPKs [63] (Figure 3d). This structural feature fits well to the fact that indeno[1,2-*b*]indole-9,10-dione, a **4p**-precurser lacking these two moieties, has an IC_50_-value for CK2 inhibition of more than 10 µM [56], that the addition of an isopropyl group at the N5-atom (resulting in the molecule visible in Figure 4a) decreases the IC_50_-value to 360 nM and that the final attachment of a prenyl alcohol substituent to the C4-atom further lowers the IC_50_ to 25 nM [52]. Remarkably, as beneficial these two substitutions are for CK2 inhibition, as unfavourable they are for the inhibition of the ABC-half transporter ABCG2, so that **4p** is not only the best known indeno[1,2-*b*]indole-based CK2 inhibitor, but also the most discriminating between the two target proteins [52].

Compared to the extensive hydrophobic embedding (Figure 3c) polar interactions contribute only marginally to the affinity of **4p** to CK2, but they are critical for its orientation within the ATP site. As illustrated in Figure 3b,d there is only one hydrogen bond of **4p** to the protein, namely from the D-ring oxo group to the side chain of Lys68 which normally serves to coordinate the α- and the β-phospho group of the cosubstrates ATP and GTP [65]. Both oxo groups of **4p** are hydrogen-bonded to HOH2 (Figure 3b,d), a conserved water molecule [43], which forms the centre of a hydrogen-bonding network connecting two water molecules, the inhibitor, Lys68, Glu81 from the helix αC and the so-called magnesium-binding loop with Asp175 as its most important component. Thus, unexpectedly—because it is contrary to a rule postulated for high-affinity CK2 inhibitors [41] and inferred from several typical cases of complex structures—**4p** does not require a hydrogen (or halogen) bond to the interdomain hinge region to reach its IC_50_-value of 25 nM [52]. 

On the other hand, this fact suggests the possibility to further optimize its CK2 affinity by introducing substituents at ring A (Figure 1a,f) suitable for anchoring to the hinge backbone. The position 1 was already subject of an SAR analysis and found to be unfavourable [52], but positions 2 and 3 of the A ring look more promising from inspection of the CK2α^1−335^/**4p** structure. 

### 2.4. Is the CK2 Binding Mode of **4p** Representative for Indeno[1,2-*b*]indole-Type CK2 Inhibitors?

An essential condition of the discovered binding mode seems to be the fact that the two oxo groups of **4p** have a perfect distance to cooperate in the formation of the critical water-mediated hydrogen-bonding network (Figure 3b,d) and thus to determine the principle orientation of the inhibitor. The presence of two potential hydrogen bond acceptors attached to C-atoms 9 (ring D) and 10 (ring B) and thus in a fixed distance is a common feature of all quinonic (Figure 1c), phenolic (Figure 1d) and ketonic (Figure 1e) indeno[1,2-*b*]indole-based CK2 inhibitors known so far. This is an argument to assume that the CK2 binding mode of **4p** might be representative for indeno[1,2-*b*]indole-type CK2 inhibitors in general.

However, the validity of this generalization is open in the moment as demonstrated by comparison with the CK2 binding mode predicted by Alchab et al. [58] for two ketonic and two quinonic CK2 inhibitors with indeno[1,2-*b*]indole scaffold. Among these, 5-isopropyl-5,6,7,8-tetra-hydroindeno[1,2-*b*]indole-9,10-dione (Figure 4a)—referred to as **5h** by Alchab et al. [58]—is the most similar to **4p**, albeit it differs from the latter by the missing of the bulky prenyl alcohol moiety attached to the A ring (Figure 1f). In order to create an *in-silico* model of the CK2α/**5h** complex Alchab et al. [58] used 3OWJ [66] as a template which is a CK2α co-crystal structure with 9-hydroxy-5,11-dimethyl-4,6-dihydro-1*H*-pyrido[4,3-*b*]carbazol-1-one (PDB ligand abbreviation “1EL”), a CK2 inhibitor with a pyridocarbazolone scaffold (Figure 4b).

The result and to a certain degree the rationale of the modelling is indicated in Figure 4c: the CK2α-bound 1EL molecule of 3OWJ was replaced by **5h** in such a way that: (i) the two annulated four-ring systems overlap maximally, (ii) the A-ring of **5h** forms a π-π-interaction with Phe113 as it is the case for the 9-hydroxybenzo group of 1EL (Figure 4b,c), (iii) the oxo substituents of the terminal non-aromatic six rings point in the same direction, namely exposed to the solvent, and (iv) that the isopropyl moiety of **5h** is buried at the hydrophobic back side of the cavity. Unlike the case of **4p** the conserved water molecules do not play an essential role for supporting this binding mode; quite the contrary, Alchab et al. [58] explicitly mention that in order to avoid sterical clashes they had to delete the hidden water molecule from 3OWJ which is equivalent to HOH2 of the CK2α^1−335^/**4p** complex and which is so critical for the ligand orientation there (Figure 3b,d). 

Taken together, following the example of the CK2α/1EL complex [66] a “hydrophobic-in/oxygen-out” mode of binding to CK2α was predicted for **5h** (and for three other CK2 inhibitors with an indeno[1,2-*b*]indole scaffold). In contrast, the reverse—a “hydrophobic-out/oxygen-in” binding mode—was found experimentally for **4p** in this study. Whether **5h** actually follows the latter and how representative it is for indeno[1,2-*b*]indole-type CK2 inhibitors in general, has to be revealed by further co-crystallization studies.

### 2.5. ***4p*** Is Not Selective with Respect to the Interdomain Hinge/Helix αD region Conformation

**Figure 5:**
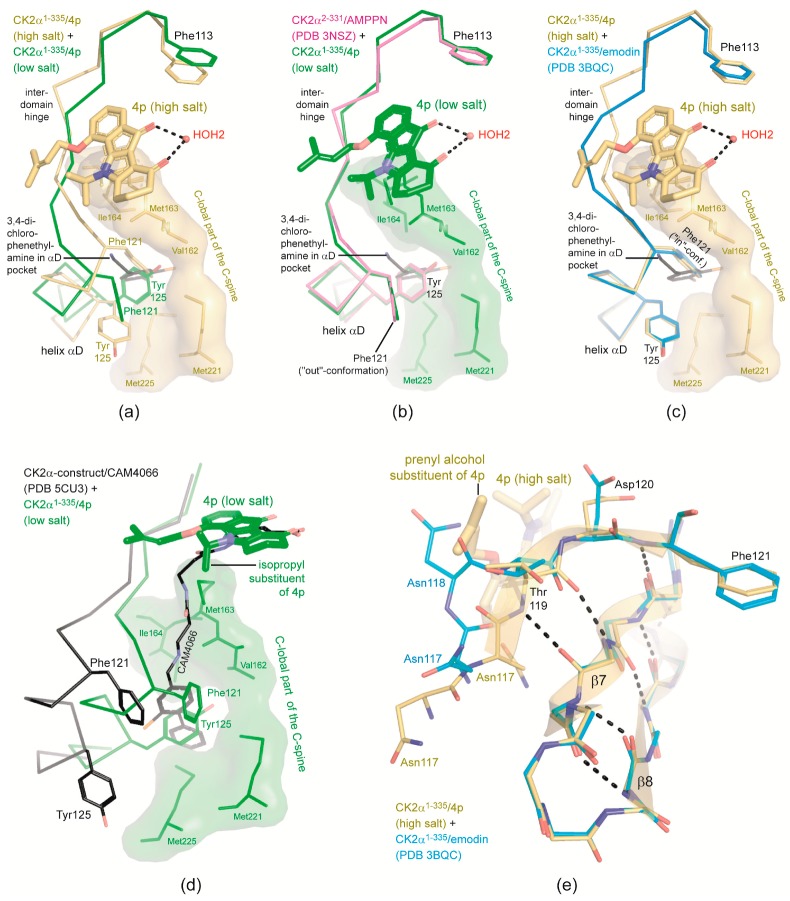
Hinge/helix αD region conformations of CK2α compatible with **4p** binding. (**a**–**c**) Pairwise comparisons of the hinge/helix αD of the high-salt and the low-salt CK2α^1−335^/**4p** structure (**a**), of the low-salt CK2α^1−335^/**4p** structure (green) and the high-resolution human CK2α structure 3NSZ (magenta) serving as a reference for the open (Phe121-out) conformation (**b**) and of the low-salt CK2α^1−335^/**4p** structure (yellow) and 3BQC (blue), a typical closed-conformation human CK2α structure (Phe121-in) (**c**). The C-terminal domain part of the catalytic spine is drawn with a surface representation from the high-salt (**a**,**c**) or from the low-salt CK2α^1−335^/**4p** structure (**b**) in order to illustrate the hydrophobic environment of the αD pocket which is indicated in all three pictures by the ligand 3,4-dichlorophenethylamine (black C-atoms) from PDB 5CLP [19]. (**d**) Overlay of the low-salt CK2α^1−335^/**4p** structure (green) with PDB 5CU3 [19] (black C-atoms), a complex structure of a human CK2α construct (with an N-terminal extension, a C-terminal deletion and the point mutation Arg21Ser) with the bivalent inhibitor CAM4066 addressing the ATP-site and the αD pocket in parallel. (**e**) Overlay of the high-salt CK2α^1−335^/**4p** structure (yellow C-atoms) with PDB 3BQC (blue C-atoms) to illustrate an extension of the antiparallel β-sheet β7/β8 (Figure 3a) by an additional β-strand established in the middle part of the hinge/helix αD region and being one residue longer in the high-salt CK2α^1−335^/**4p** complex than in the CK2α^1−335^/emodin complex. β-sheet-typical H-bonds in the CK2α^1−335^/**4p** complex are depicted as dotted lines. For reasons of clarity no side chains are drawn for the residues of the strands β7 and β8.

In order to explore if the crystallization conditions—in particular the dominance of a kosmo-tropic salt supporting hydrophobic interactions—affect the local protein conformation or even the orientation of the inhibitor as observed in previous CK2α/inhibitor complexes [38,62,67], we superimposed the CK2α^1−335^/**4p** crystal structures resulting from low- and from high-salt crystallization conditions (structure no. 1 and 2 of Table 1). While the water-anchored **4p** molecules bound to the ATP sites are very similar (compare Figure 5a with Figure 5b), the comparison reveals large conformational differences for the enzyme matrices in the interdomain hinge/helix αD region (Figure 5a): (i) under low-salt conditions this region of the CK2α^1−335^/**4p** complex adopts a state found in the majority of CK2α-structures and known as “open” or “Phe121-out” conformation [68]; the local structure here is almost identical to PDB entry 3NSZ [44], a prototypical CK2α complex structure with an ADP analog provided with an open hinge/helix αD region (Figure 5b); (ii) in contrast under high-salt crystallization conditions the “closed” or “Phe121-in” conformation of the hinge/helix αD region known for instance from the CK2α^1−335^/emodin complex (PDB 3BQC [46]) can be identified (Figure 5c) albeit with significant differences in the hinge part as perceptible from Figure 5c and as discussed below. This closed state was observed so far only in some structures of human CK2α although it is the canonical conformation of EPKs in the hinge/helix αD region [69,70]. Obviously, **4p** is not conformationally selective with respect to the hinge/helix αD area which fits to the aforementioned fact that it does not form any polar interaction with that part of the enzyme.

Recently, even further distinct conformations were detected for the hinge/helix αD region [19,38], one of them being accompanied with a novel small molecule binding site next to the αD helix [19]. This “αD pocket”—in Figure 5a-c indicated by the 3,4-dichlorophenethylamine ligand from the PDB structure 5CLP [19]—can be exploited for the generation of highly selective bivalent CK2 inhibitors [19,20] like CAM4066 (Figure 5d). To couple **4p** or another indeno[1,2-*b*]indole-type CK2 inhibitor to an αD pocket ligand in order to create a bivalent inhibitor with improved selectivity is an attractive option; it is supported first by the modest nature of **4p** with respect to the hinge/helix αD conformation suggesting that access to the αD pocket parallel to the binding of an indeno[1,2-*b*]indole-type ligand might be possible and second by **4p**’s orientation within the ATP site with the two organic substituents at the surface and in particular with the isopropyl group pointing in the direction of the putative αD pocket (Figure 5d).

The conformational plasticity of the hinge/helix αD zone in human CK2α is already known since 2005 [71]. The late discovery of the αD pocket [19] resulted from the fact that in all previous CK2α structures it was occupied by a side chain from the helix αD itself: either by Tyr125 in the open hinge/helix αD conformation (Figure 5a,b) or by Phe121 in the closed one (Figure 5a,c) where it completes a hydrophobic cluster referred to as the “catalytic spine” (C-spine) [72]. Generally in EPKs, the C-spine and the accompanying “regulatory spine” (R-spine) are stacks of hydrophobic residues that connect the two main domains of the kinase core and that are required for full activity [72]. Typically, crystallization conditions dominated by high concentrations of a kosmotropic salt support hydrophobic interactions. Therefore, they are particularly promotive for the closed hinge/helix αD conformation in which the hydrophobic C-spine is fully assembled. This rule originally derived from a survey of all human CK2α crystallization studies published at that time [67] is further confirmed here by the high-salt CK2α^1−335^/**4p**-structure with Phe121 being embedded in the C-spine environment (Figure 5a,c).

Remarkably, the propensity to optimize the hydrophobic C-spine cluster under high-salt conditions is strong enough here to overcome an opposing restraint imposed by the bound inhibitor: normally, CK2α ligands like **4p** that require the hydrophobic region II for binding (Figure 3d) are not compatible with the closed hinge/helix αD conformation because this conformation as it is known so far (exemplified by 3BQC [46] in Figure 5c,e) is occupied by parts of the enzyme itself (e.g., Asn118 in Figure 5e) [68]. Here, such spatial problems are avoided in a structurally most elegant way: as described earlier [68] the closed hinge/helix αD conformation is supported by the establishment of a short β-strand ranging from Thr119 to Phe121, thus extending the small antiparallel β-sheet formed by the strands β7 and β8 (Figure 3a). In the high-salt CK2α^1−335^/**4p** structure this additional β-strand is enlarged to comprise now the zone from Asn118 to Phe121. This brings the Asn118 side chain out of conflict with the prenyl alcohol substituent of **4p** (Figure 5e). Significant conformational backbone adaptations of Asn117 and Asn118 are required to this end, but asparagine is well suited for that task because within folded proteins it is the most versatile amino acid after glycine with respect to the conformational space occupied by the backbone torsion angles φ and ψ [73].

### 2.6. Structural Characteristics of the CK2α′^Cys336Ser^/***4p*** Complex

**Figure 6:**
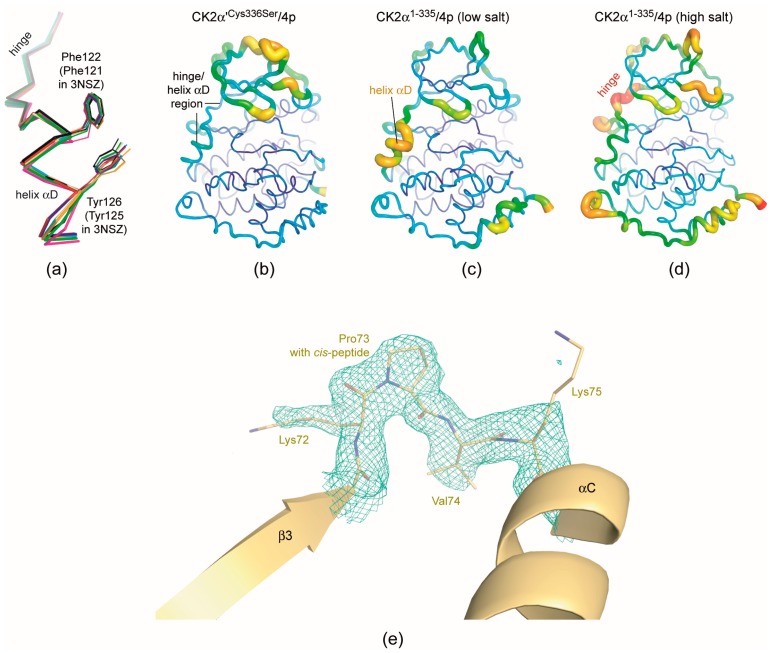
Structural features of the CK2α′^Cys336Ser^/**4p** complex. (**a**) Overlay of the hinge/helix αD regions of the two CK2α′ protomers of this study (red and blue), of CK2α^2−331^ in 3NSZ [44] as a reference structure for the open hinge/helix αD conformation (black) and of all other CK2α′ chains available in the PDB (3OFM [31]: green; 3E3B [39]: magenta; 5M4U [38]: orange; 5M56-chain A: cyan; 5M56-chain B [38]: yellow); due to an insertion of one residue in the N-terminal segment of CK2α′ the numbering schemes of CK2α and CK2α′ are not identical: Phe121 and Tyr125 of CK2α are equivalent to Phe122 and Tyr126 of CK2α′. (**b**–**d**) Rainbow representation of the atomic B-factors in the CK2α′^Cys336Ser^/**4p** complex (**b**) and for comparison in the low-salt the CK2α^1−335^/**4p** complex (**c**) and in the low-salt the CK2α^1−335^/**4p** complex (**d**). Large B-factors (and thus high internal mobilities): red and thick; low B-factors (and thus low internal mobilities): blue and thin. (**e**) A cis-peptide bond between Lys72 and Pro73 in chain A of the CK2α′^Cys336Ser^/**4p** complex structure testified by the final electron density (cutoff level 1.0 σ).

While **4p** binding in both chains of the CK2α′^Cys336Ser^/**4p** complex is essentially identical to what is seen in CK2α^1−335^, the CK2α′^Cys336Ser^/**4p** complex structure (no. 3 of Table 1) a few additional remarks are warranted:

(i) Like previous structures of full-length human CK2α′ [31,38] the C-terminal segment comprising almost 20 residues and being completely unrelated to human CK2α with respect to the primary sequence is not visible in the electron density due to inherent flexibility.
(ii) As mentioned in the last section structural plasticity or even “hypervariability” of the hinge/helix αD region—casually accompanied by the occurrence of the αD pocket [19]—is a significant feature of human CK2α, but it was never observed so far in structures of maize or yeast CK2α and human CK2α′ where the hinge/helix αD region was always found in the open conformation without any exception. Correspondingly, crystals grown under high-salt conditions were never described for those CK2α homologs. The CK2α′^Cys336Ser^/**4p** complex of this study confirms these experiences: neither do the two CK2α′^Cys336Ser^ protomers deviate from the open hinge/helix αD conformation (Figure 6a) nor did we observe any crystallization hit under high-salt conditions.
  The lack of any conformational ambiguity in the hinge/helix αD region of the CK2α′^Cys336Ser^/**4p** complex is also perceptible from the final atomic B-factors which reflect the mobilities of the atoms in the crystalline state: they are low in the whole hinge/helix αD area of the CK2α′^Cys336Ser^/**4p** complex (Figure 6b) while in both CK2α^1−335^/**4p** complex structures high mobility sections exist, namely either at the helix αD (low-salt structure; Figure 6c) or at the hinge (high-salt structure; Figure 6d). For maize CK2α the fixation to the open hinge/helix αD conformation was plausibly explained with restraints imposed by a proline residue at the C-terminal end of helix αD instead of Gln126 in human CK2α [70]. In the case of human CK2α′, however, no equivalent exchange exists. Rather, the sequences of the two human paralogs in this region are so similar that no particular enzyme-inherent restraints in favour of the open conformation are evident. Insofar, it is an open question whether in future CK2α′ structures the open hinge/helix αD conformation will prevail as well. For inhibitor development, it is even more interesting if CK2α′ conformations with an αD pocket accessible for small molecule exists at all. If not, inhibitors addressing the αD pocket should be selective for human CK2α over CK2α′.
(iii) Finally, the CK2α′^Cys336Ser^/**4p** complex structure provides a further case of a *cis*-proline (Pro73) residue in the β3/αC loop (Figure 6e). As in previous instances of this phenomenon—a CK2α^1−335^ complex structure with a CK2β-competitive cyclic peptide (PDB 4IB5) [74] and a CK2α′^Asp39Gly/Cys336Ser^ complex structure with the flavonol-derived inhibitor FLC21 (PDB 5M56) [38]—this peptide switch occurs only in one of two (5M56) or three (4IB5) protomers in the asymmetric unit, namely in chain A while in chain B the Lys72/Pro73 peptide has the normal *trans*-configuration. The Lys/Pro dipeptide in the β3/αC loop is absolutely conserved in the sequences of CK2α homologs, but it is completely unknown so far under which conditions a *cis*-peptide bond can be trapped within this dipeptide and whether a functional relevance is associated with this feature.

## 3. Materials and Methods

### 3.1. CK2 Inhibitor

The CK2 inhibitor **4p** was synthesized as described by Gozzi et al. [52].

### 3.2. Caco-2 Cell Permeability Assay

Human epithelial colorectal adenocarcinoma cells (Caco-2) were used to elucidate membrane permeability of compound **4p**. Caco-2 cells were cultured using Dulbecco’s modified Eagle’s medium (DMEM) with the addition of 1% (*v*/*v*) non-essential amino acids, 1% (*v*/*v*) penicillin/ streptomycin/glutamine, and 10% fetal calf serum (FCS) in a humidified chamber at 5% CO_2_ and 37 °C. For the permeability assay 60,000 cells were seeded on transwell filters (Transwell^®^, 12 well plate, Corning^®^, Corning, NY, USA) and were cultivated for 3 weeks. The integrity of the monolayer was determined using the Transepithelial Electrical Resistance (TEER). Medium in the apical compartment was removed and was replaced by fresh medium containing compound **4p** in a concentration of 100 µM in 1% DMSO. Rhodamine B (10 µg/mL) served as a control for a permeable substance and FITC-Dextran-4 (10 µg/mL) as a control for non-permeable substance. Caco-2 cells were incubated for 24 h at 37 °C and 5% CO_2_. TEER values were monitored every 20 min. After 24 h the concentrations of compound **4p** as well as control substances were determined in the basolateral compartment.

Permeation of the control substances were evaluated by measuring the emitted fluorescence at 627 nm for Rhodamine B (excitation at 554 nm) and at 528 nm for FITC-Dextran-4 (excitation at 485 nm). RP-HPLC (EC 125/4 Nucleodur C18 Htec, Macherey-Nagel, Düren, Germany) was used to measure the concentration of **4p** in the basolateral samples. Before separation the samples were purified using solid phase extraction (Chromafix C_18_ec, Macherey-Nagel) following the procedure of Toth et al. [75]. Twenty μL of the purified sample was injected into the RP-HPLC. The chromatography was performed with a flow rate of 0.5 mL/min, with a run time of 17 min, at a temperature of 40 °C, with UV monitoring at 250 nm and with a gradient mobile phase ranging from 10% (*v*/*v*) CH_3_CN in H_2_O with 0.05% trifluoro acetic acid to 90% (*v*/*v*) CH_3_CN in H_2_O with 0.05% trifluoroacetic acid.

The apparent permeability coefficient (Papp) was calculated using the following equation published by Artursson et al. [60]:

$$P_{\mathrm{app}}{\;=\;V}_{b\;}\times \;\frac{c_{b}}{t}\;\times \;\frac{1}{c_{0}\;\times \;A}$$

In this equation V_b_ is the volume of the recipient (basolateral) compartment, c_b_ is the concentration of the compound in the basolateral compartment, t is the incubation time, c_0_ is the initial concentration of the compound in the donor (apical) chamber and A is the membrane surface area.

### 3.3. Protein

CK2α^1−335^, the C-terminally truncated version of human CK2α, was expressed and purified as described previously [61], with the exception that the initial P11 phosphocellulose chromatography run was replaced by a batch purification step using the same resin material.

The construct of the full length CK2α′ has an N-terminal His_6_-tag and carries the single C-terminal point mutation Cys336Ser (CK2α′^Cys336Ser^). Recombinant CK2α′^Cys336Ser^ was expressed in *E. coli* C41(DE3) cells after induction with 0.5 mM isopropyl-β-d-thiogalactopyranoside for 3 h at 37 °C and purified as described by Guerra et al. [76]. 

Purified CK2α^1−335^ and CK2α′^Cys336Ser^ were rebuffered in storage solution (25 mM Tris/HCl, 500 mM NaCl, pH 8.5). The final concentrations were 15.0 mg/mL for CK2α^1−335^ and 11.0 mg/mL for CK2α′^Cys336Ser^ as determined via UV-absorption at 280 nm.

### 3.4. Crystallization

CK2α^1−335^ was diluted with storage solution to a concentration of 6.0 mg/mL. Ninety μL of the protein solution were mixed with 10 μL of a 10 mM **4p** solution in DMSO. After incubating the mixture for 30 min at room temperature precipitate was removed by centrifugation. CK2α^1−335^/**4p** co-crystals were obtained in a low- and a high-salt condition using the sitting drop variant of vapor diffusion at 20 °C. The optimized drop composition was 2 μL of the preincubated CK2α^1−335^/**4p** mixture and 1 μL of reservoir solution. The optimized reservoir solutions were 25% (*w*/*v*) PEG5000, 0.2 M ammonium sulfate, 0.1 M MES, pH 6.5, (low-salt condition) and 4.2 M NaCl, 0.1 M citric acid, pH 5.5 (high-salt condition).

In the case of CK2α′^Cys336Ser^ the procedure was similar: the enzyme solution was diluted with storage buffer to a concentration of 5.0 mg/mL. Ninety μL of the resulting protein solution were mixed with 10 μL of a 10 mM **4p** solution in DMSO. After 30 min incubation of the mixture at room temperature, precipitates were removed by centrifugation. CK2α′^Cys336Ser^/**4p** co-crystals grew in condition G8 of the Index^TM^ crystal screen (Hampton Research, Aliso Viejo, CA, USA) which is composed of 25% (*w*/*v*) PEG3350, 0.2 M ammonium acetate, 0.1 M HEPES, pH 7.5. No CK2α′^Cys336Ser^/**4p** crystals grew under high-salt conditions. To obtain CK2α′^Cys336Ser^/**4p** crystals suitable for X-ray diffractometry micro-seeding had to be applied.

### 3.5. X-ray Diffraction Data Collection and Processing

To prepare X-ray diffraction data collection at a temperature of 100 K CK2α^1−335^/**4p** and CK2α′^C336S^/**4p** co-crystals were flash frozen in liquid nitrogen. Cryo conditions for the CK2α^1−335^/**4p** crystals grown under low-salt conditions were achieved by adding 0.2 μL butane-1,3-diol into the drop. The high-salt crystallization condition of the CK2α^1−335^/**4p** complex was already under cryo conditions. In the case of the CK2α′^C336S^/**4p** crystals cryo conditions were obtained by incubating the crystals for 1 min in a solution containing PEG3350 at its solubility limit, 30% (*v*/*v*) ethylene glycol, 0.2 M ammonium acetate, 0.1 M HEPES, pH 7.5.

X-ray diffraction data were collected at various synchrotron beamlines: at beamline X06DA of the Swiss Light Source (SLS) in Villigen (Switzerland) with high-salt CK2α^1−335^/**4p** crystals, at beamline ID30a-1 of the European Synchrotron radiation facility (ESRF) in Grenoble (France) with low-salt CK2α^1−335^/**4p** crystals, and at beamline P13 of the EMBL outstation at the Deutsches Elektronensynchrotron (DESY) in Hamburg (Germany) with CK2α′^C336S^/**4p** crystals. The wave-lengths used for diffractometry are given in Table 1. All diffraction data were processed with XDS [77] for integration followed by POINTLESS and AIMLESS [78] for symmetry determination and scaling and CTRUNCATE from the CCP4 suite [79] for calculation of the structure factor amplitudes.

### 3.6. Structure Solution, Refinement, Validation, Deposition and Illustration

The structures were solved by molecular replacement with PHASER [80] using the protein chain of the CK2α^1−335^ structure 2PVR [1] as a search model for the CK2α^1−335^/**4p** complexes and 3OFM ^31^ for the CK2α′^Cys336Ser^/**4p** complex. The structures were refined and validated with PHENIX [81] which was also used to generate the topology of the **4p** molecule. Manual corrections of the structures were performed with COOT [82]. The final structures are available at the Protein Data Bank [57] under the accession codes 5OMY (high-salt CK2α^1−335^/**4p** structure), 5ONI (low-salt CK2α^1−335^/**4p** structure) and 5OOI (CK2α′^Cys336Ser^/**4p** structure). Figure 3b was drawn with LIGPLOT [83]; all other structure illustrations were prepared with PYMOL [84].

## 4. Conclusions

The indeno[1,2-*b*]indole scaffold is a versatile and valuable lead structure to develop inhibitors targeting cancer associated proteins like protein kinase CK2 [50,51], the breast cancer resistance factor and ABC half transporter ABCG2 [52] and the phosphatase CDC25 [55]. Such efforts strongly benefit from experimental structure information about protein/inhibitor interactions which is presented here for the first time for an indeno[1,2-*b*]indole-type CK2 inhibitor and which can be exploited for further optimization in the future. Most importantly, the crystal structures of this molecule with either human CK2α or its paralogous isoform CK2α′ revealed a feature difficult to predict *in-silico*, namely a hidden water molecule as a critical determinant of the inhibitor orientation.

## Figures and Tables

**Table 1:** Crystallization, X-ray diffraction data and refinement statistics.

Structure No.		1	2	3
PDB Code		5OMY	5ONI	5OOI
Crystallized Complex		CK2α^1−335^ + **4p**		CK2α^Cys336Ser^′ + **4p**
*Crystallization*				
Vapour diffusion reservoir composition		4.2 M NaCl, 0.1 M sodium citrate, pH 5.5	25% (*w*/*v*) PEG5000, 0.2 M ammonium sulfate, 0.1 M MES, pH 6.5	25% (*w*/*v*) PEG3350, 0.2 M ammonium acetate, 0.1 M HEPES, pH 7.5
Sitting drop composition before equilibration		1 μL reservoir + 1 μL enzyme/**4p** mixture (90 μL 5 mg/mL enzyme, 0.5 M NaCl, 25 mM Tris/HCl, pH 8.5, mixed and pre-equilibrated with 10 μL 10 mM **4p** in DMSO)		1 μL reservoir + 1 μL enzyme/**4p** mixture (90 μL 5 mg/mL enzyme, 0.5 M NaCl, 25 mM Tris/HCl, pH 8.5, mixed and pre-equilibrated with 10 μL 10 mM **4p** in DMSO)
***X-ray Diffraction Data Collection***				
Wavelength [Å]		0.97625	0.9660	1.0000
Synchrotron (beamline)		SLS (X06DA)	ESRF (ID30A-1)	PETRA III at DESY (P13)
Space group		P4_3_2_1_2	P4_3_2_1_2	P2_1_2_1_2_1_
Unit cell	a, b, c [Å]	72.70, 72.70, 132.89	128.45, 128.45, 124.11	46.49, 112.13, 143.69
	α, β, γ [°]	90.0, 90.0, 90.0	90.0, 90.0, 90.0	90.0, 90.0, 90.0
Protomers per asym. unit		1	2	2
Resolution [Å] (highest resolution shell)		63.78–1.95 (2.02–1.95) ^1^	57.04–2.00 (2.072–2.00) ^1^	60.49–2.00 (2.07–2.00) ^1^
R_sym_ [%]		9.2 (228.1) ^1^	9.3 (65.7) ^1^	17.7 (119.1) ^1^
CC1/2		0.999 (0.640) ^1^	0.996 (0.685) ^1^	0.995 (0.673) ^1^
Signal-to-noise ratio (I/σ_I_)		20.77 (1.52) ^1^	14.20 (0.93) ^1^	7.39 (1.41) ^1^
No. of unique reflections		26747 (2639) ^1^	70221 (6881) ^1^	51742 (4979) ^1^
Completeness [%]		99.94 (100.0) ^1^	99.51 (98.84) ^1^	99.69 (97.59) ^1^
Multiplicity		24.9 (26.1)^1^	6.2 (6.3) ^1^	6.7 (6.7) ^1^
Wilson B-factor [Å^2^]		43.54	44.40	24.98
***Structure Refinement***				
No. of reflections for R_work_/R_free_		25417/1316	68727/1388	50784/1041
R_work_/R_free_ [%]		19.25/22.08	17.51/20.42	17.37/22.10
Number of non-H-atoms		2966	6055	6050
Protein		2812	5645	5524
Ligand/Ion		32	113	83
Water		122	297	443
Average B-factor [Å^2^]		56.42	55.51	34.15
Protein		56.63	55.10	33.80
Ligand/Ion		64.83	79.16	34.33
water		49.51	54.20	38.55
RMS deviations				
Bond lengths [Å]		0.003	0.007	0.011
Bond angles [°]		0.570	0.81	1.13
Ramachandran plot				
favoured (%)		96.36	96.99	97.38
allowed (%)		3.64	3.01	2.46
outliers (%)		0.30	0.00	0.15

^1^ Values in brackets refer to the highest resolution shell.
